# Endocrine-Disrupting Chemicals in Human Fetal Growth

**DOI:** 10.3390/ijms21041430

**Published:** 2020-02-20

**Authors:** Maria Elisabeth Street, Sergio Bernasconi

**Affiliations:** 1 Department of Mother and Child, 42123 Azienda USL–IRCCS di Reggio Emilia, Italy; mariaelisabeth.street@ausl.re.it; 2 Microbiome Research Hub, University of Parma, Via A. Catalani 10, 43123 Parma, Italy

**Keywords:** endocrine-disrupting chemicals (EDCs), pregnancy, preterm birth, intrauterine growth restriction (IUGR), small for gestational age (SGA), fetal growth restriction (FGR), birth weight, placenta, postnatal outcomes, developmental origins of health and disease (DOHAD)

## Abstract

Fetal growth is regulated by a complex interaction of maternal, placental, and fetal factors. The effects and outcomes that chemicals, widely distributed in the environment, may have on the health status of both the mother and the fetus are not yet well defined. Mainly mixtures of chemical substances are found in the mothers and placenta. Exposure to endocrine-disrupting chemicals (EDCs) can be associated with fetal growth retardation, thyroid dysfunction, and neurological disorders. EDCs mostly interfere with insulin, glucocorticoid, estrogenic, and thyroid pathways, with subsequent effects on normal endocrine and metabolic functions, which cause changes in the epigenome and state of inflammation with life-long effects and consequences. International scientific societies recommend the implementation of research and of all possible preventive measures. This review briefly summarizes all these aspects.

## 1. Background

Fetal growth is regulated by a complex interaction of maternal, placental, and fetal factors, which remain partially known.

Maternal intake of the correct amounts of nutrients (carbohydrates, lipids, proteins, minerals, vitamins) and oxygen certainly plays a fundamental role, as demonstrated by the altered fetal growth observed both in humans and in other animal species if decreased or excessive intake of these substances occurs [1]. However, it should be kept in mind that fetal growth largely depends on endocrine factors, and nutritional deficiency or excess determines important variations [1].

Among the various hormones involved (thyroid hormones, insulin, Growth hormone (GH) variants, leptin, cortisol) a key role is played by Insulin-like growth Factor (IGF)-1 and IGF-2 [2], and it is important to cite (see the following chapters) that the inflammatory and the IGF systems also interact [3]. In recent years, additional factors were discovered and proved to be of upmost importance [4,5,6] such as Activin, A.; Human corionic gonadotropin (HCG), and retinoic acid and its receptors in animal models [7].

It is known for a long time that various stimuli can modify the homeostasis of the systems that regulate growth in the uterus, increasing the risk of pathological states. Maternal illnesses (like infections, diabetes, or autoimmune diseases) and/or bad mother’s lifestyle habits (tobacco, alcohol, drugs) and/or the use of therapeutic drugs (antiepileptic drugs) can cause a derangement in fetal growth.

More recently, many researchers began studying more deeply the outcomes that chemicals, widely distributed in the environment, may have on the health status of both the mother [8] and the fetus [9].

Therefore, this short review focuses on the most recent aspects and knowledge relative to exposure to known endocrine-disrupting chemicals (EDCs) and their effects on pregnancy, placenta, and fetus, and it is intended as a reflection for future research purposes, and not as a systematic review of the literature. For this purpose, we searched for publications over the last 15 years in PubMed using the following words for the search: endocrine-disrupting chemicals (EDC), endocrine disruptors, pregnancy, preterm birth, intrauterine growth restriction (IUGR) and fetal growth restriction (FGR), neonatal size, large and small for gestational age (LGA and SGA, respectively), birth weight, placenta, postnatal outcomes, and developmental origins of health and disease (DOHAD). Animal and in vitro studies were not taken into consideration, nor were the effects of EDCs on fertility.

The main aims of this minireview are (1) to provide an update of what we know about the relationships among endocrine-disrupting chemicals (EDCs), which are described as human-made substances that alter hormone regulation in humans or wildlife [10], pregnant women’s health, and fetal and postnatal growth and metabolism, and (2) to highlight why a new research approach is needed, based on new technologies, statistics, and a more holistic vision, to comprehensively understand mechanisms of disease that originate in utero.

## 2. From Environment to Pregnant Mother

The presence of EDCs in pregnant women was the subject of many studies. Many focused on **single chemicals**.

For example, phthalate metabolites (plasticizers present in personal care products, textiles, food packaging) were measured mainly in urine, collected at different times of pregnancy, and they were found above the limit of detection (LOD) for these substances in at least one urine sample in 50% of the women [11]. Triclosan (TCS) and triclocarban (TCC), which are present in a wide variety of consumer products like soaps, toothpaste, medical devices, plastics, and textiles, were measured in urine samples, and TCS was detected in all participants of one study [12].

Paraben metabolites (widely used for a long time as a preservative in commonly used products) were recently discovered to act as endocrine disruptors particularly in children, and they were found in up to 100% of the maternal urine samples examined [13].

Moreover, other studies dealt with **mixtures of chemical substances.**

For example, polychlorinated biphenyls, organochlorine pesticides, phenols, perfluorinated compounds, polybrominated diphenyl ethers, phthalates, polycyclic aromatic hydrocarbons, and perchlorate were detected in 99%–100% of samples from pregnant women. The median number of detected chemicals by chemical class ranged from 4–12 perfluorinated compounds and from 9–13 phthalates [14].

Variations in the levels of EDCs were observed in relation to the season [15], ethnic groups [13], type of work performed [16], and socio-economic conditions [17]. As a demonstration of the global environmental diffusion of EDCs, it was also shown that their presence may not differ between pregnant and non-pregnant women [14].

In recent years, many authors paid more attention to the role that the placenta can play in determining the health of the mother and fetus in the short and long term, and they called for more research in this field [18,19].

In this context, researchers are studying the possible effects that EDCs can cause at the placental level [20], and some of the most important knowledge that emerged so far is summarized below.

Different EDCs were found in the placenta (pesticides, personal care products, polybrominated diphenyl ethers, bisphenol, A.; polychlorinated biphenyls), and their concentration could be associated with fetal growth restriction, thyroid dysfunction, and neurological disorders [21]. Phthalates not only cross the feto-placental barrier but disrupt placental growth and development in pregnant mice [22]. In humans, Zhu et al. [23], studying mother urinary phathalate excretion in the three trimesters of pregnancy, suggested that prenatal phthalate exposure was associated with altered placental size and shape, and that exposure to certain phthalates could induce the placenta to become thicker and more circular. Moreover, the associations observed appeared stronger in the males. This latter observation could be in agreement with the hypothesis that the placenta seems to be a major contributor to the sexual dimorphism of diseases [24]. Disruption of normal placental growth was also reported in association with the exposure of polybrominated diphenyl ethers (PBDEs; flame retardants which are used globally in the manufacture of electronic equipment, furniture, textiles, foam, and household plastic products) measured in umbilical blood samples [25].

The mechanisms via which EDCs act on the placenta and modify its morphology and function are largely unknown. However, some studies put forward a few hypotheses. Thyroid hormones are among the key regulators of placental development, and their actions are mainly mediated by the thyroid hormone receptors (THRs) encoded by the Thrα1 and Thrβ1 genes. Phthalate exposure in animal models determines both Thrα1 and Thrβ1 gene downregulation and inhibition of nuclear translocation in the placenta [26]. Furthermore, exposure to polycyclic aromatic hydrocarbons (PAHs) and polybrominated diphenyl ethers (PBDEs) modifies IGF-1 placental content [27]. Recent research increasingly shows that DNA methylation is sensitive to environmental exposure, and this is able to influence the human placental methylome, causing aberrant placental development and function, thus influencing fetal outcomes [28]. Many environmental factors (smoking, air pollution, cadmium) are able to do this, including the following EDCs:

**Phthalates:** Exposure to phthalates can alter gene methylation and expression, with epidermal growth factor receptor (EGFR) being one of the critical genes mediating effects on early placental function [29]. Urinary concentrations of mono (2-ethyl-5-hydroxyhexyl) phthalate (MEHHP), and mono (2-ethyl-5-oxohexyl) phthalate (MEOHP) were observed to correlate negatively with placental IGF-2 DNA methylation, particularly in fetal growth restriction (FGR) newborns [30]. IGF-2 is a major regulator of placental and fetal growth [31].

**Persistent organic pollutants (POPs):** Although their production and use were long prohibited, these substances are still widely distributed in the environment due to their persistence. Exposure to several POPs including Para-Dichlorodiphenyltrichloro ethane (DDTs) is associated with changes in gene methylation including the IGF-2 gene [31].

**Bisphenol-A (BPA):** Merck et al. [5] showed that BPA is transported through the placenta and increases β-HCG, thus affecting placental growth [6]. In following studies, others proved that, in the first trimester of pregnancy, the amount of BPA in trophoblast cells was proven to interfere with cellular growth and to affect DNA methylation [32].

In conclusion, whereas it is clear that exposure to endocrine factors before and during pregnancy has effects on placental growth and development with subsequent effects on fetal growth, many endocrine disruptors are yet unknown, as well as the mechanisms via which they disrupt. In humans, few endocrine pathways were studied to date, and little is known on the epigenetic effects. Many pathways and factors also remain unknown within normal physiology. It is clear that this field requires new research and new research approaches in order to achieve knowledge on subsequent human disease.

## 3. From Placenta to Newborn

The knowledge on how EDCs pass through the placental barrier is still scarce. On the one hand, we have evidence of a passive passage [33]; however, on the other hand, it seems that specific transport mechanisms are involved, probably depending on the specific EDC [34]. For instance, differences in polar groups and lengths of alkylated side chains seem to play an important role in materno-fetal transport of PFAAs (perfluoroalkyl substances) [35]. Once the EDCs reach the fetus, they become distributed in the different tissues [36]. Furthermore, it was reported that socio-economic factors such as maternal age can influence placental transfer [37].

It is known for a long time that low birth weight (LBW) (defined according to the World Health Organization (WHO) as a birth weight below 2500 g) and prematurity (birth occurring before the 37th week of gestation) can increase postnatal mortality and infant morbidity. Moreover, on the basis of the Developmental Origins of Health and Disease (DOHaD) hypothesis, these aspects play an important role in risk during adulthood to develop diseases such as hypertension, stroke, coronary heart disease and related disorders, and diabetes [38]. Based on these considerations, over the last few years, research strove to understand whether EDCs can affect duration of pregnancy and birth weight.

The possible influence of EDCs on the duration of pregnancy was studied, but the results are conflicting. According to Pergialiotis et al., pregnant women with high exposure to BPA are more likely to deliver preterm [39] but other authors disagreed [40]. The conclusions of studies evaluating the impact of phthalate exposure on the duration of pregnancy are also inconsistent; some of these claimed there was no effect [41], while others reported either decreased [42] or increased gestational age at delivery [43].

The results of studies on birth weight, obtained by evaluating the most widespread EDCs in the environment, can be summarized as follows:

**Bisphenol-A:** An extensive review of the literature was performed by Zee et al. [36] and meta-analyses were performed by Pergialiotis et al. [39] and Hua et al. [44]. However, it is difficult to draw definite conclusions. Hua et al. [44] concluded that prenatal BPA exposure was not significantly associated with birth weight, whereas Zee et al. [40] came to opposite conclusions and reported a negative relationship between increased BPA concentrations and a low birth weight, whereas Pergialiotis et al. [39] underlined that a significant number of studies showed a negative correlation between birth weight and exposure, although possibly not clinically relevant, as the average difference observed was around 100–200 g and, thus, insufficient to define clearly possible changes in fetal growth. Moreover, it remains unclear whether males or females are the most vulnerable. In addition to the previous data, a strong negative association between maternal preconception BPA levels and offspring birth size, among a sub-fertile population, was reported; it was hypothesized that germ cells were mediating these effects, possibly through epigenetic modifications both in oocytes and in spermatozoa, and that these changes were inherited by the offspring [45].

**Phthalates:** Studies investigating the relationship between phthalate exposure and birth weight did not lead to definitive conclusions. The results are indeed very heterogeneous as previously pointed out by Zee et al. in his review [40]. More recently, some studies suggested a positive association between in utero phthalate exposure and low birth weight [46,47], while others did not [43,48,49].

**PFAAs:** Perfluoroalkyl acids (PFAA) are used in a wide variety of industrial and consumer applications, due to their water- and lipid-repellent properties. The two most widely used PFAAs are perfluorooctanoic acid (PFOA) and perfluorooctane sulfonic acid (PFOS). A systematic review on the toxicological and epidemiological evidence of the effects of PFOA on fetal growth was published in 2014, based on the work of a team with expertise in the field of systematic reviews, environmental health, epidemiology, biology, statistics, and risk assessment [50], and it concluded that “*there is “sufficient*” human evidence that developmental exposure to PFOA reduces fetal growth. Three years later, however, Negri et al. [51], further reviewing the literature and using a specific method combining toxicological and epidemiological evidence, reported that PFOA and PFOS had a negative effect on birth weight both in humans and rodents; however, the extrapolated serum concentrations in the rodents were 102–103 times higher than those in humans, thus reducing the “*biological plausibility of a causal relationship*”.

**Polybrominated diphenyl ethers (PBDEs):** Few epidemiological studies were carried out relative to the possible relationship between additive flame retardants (present in electrical equipment, textiles, and other materials) [52], detected in 97% of the United States of America (USA) population [53], and birth weight. In this case, results were also discordant. Serme-Gbedo et al. [54] evaluated nine epidemiological studies assessing the relationship between PBDEs and birth weight, and four reported a negative association, while two reported a non-significant negative association, two others reported no statistically significant association, and one reported a negative association for male infants only, and a positive association for female newborns only.

In conclusion, it is not surprising that, considering the effects on EDCs on the placenta, the duration of pregnancy and birth weight might be affected; however, conclusive studies are still missing, and studies are primarily necessary to clarify this endpoint.

## 4. EDCs in Pregnancy and Postnatal Consequences

Research relative to the exposure/presence of EDCs during fetal life and post-natal outcome is of extreme interest and is yet limited. Further careful studies, carried out with appropriate methodologies, are worthwhile both for the fundamental importance of this field and for the uncertainty of current knowledge. For example, Ejaredar et al. (2015), in a review of the literature carried out on nine published papers, reported how the outcome was different in boys and girls depending on the exposure during pregnancy to low-molecular-weight (LMW) or high-molecular-weight (HMW) phthalate metabolites; adverse cognitive and behavioral outcomes were more frequent in boys if exposed during pregnancy to LMW, and in females if exposed to HMW metabolites [55]. On the contrary, Kim et al., examining six-year-old children, reported that childhood and not maternal phthalate exposure was related with a negative effect on Quotient of Intelligence (IQ) and attentional performance [56]. Moreover, the correlation between phthalate exposure and development also seems to be more important during postnatal life (particularly at three years of age) in longitudinal studies carried out from pregnancy onwards [57]. In a more recent meta-analysis, phthalate exposure (obtained by measurement of urinary DHEP (di-(2 ethylhexyl) phthalate)) was again reported to be more dangerous during childhood than during fetal life. Specifically, a two-fold increase during childhood was associated with a 0.8-point reduction in IQ, and a two-fold increase in prenatal maternal urine was associated with a 0.6-point reduction in the psychomotor developmental index (PDI) [58].

## 5. Pathogenetic Mechanisms

The mechanisms via which EDCs can interfere with normal endocrine homeostasis are manifold and were recently widely reviewed [59,60,61]. Summarizing, EDCs mainly interfere with insulin, glucocorticoid, estrogenic, and thyroid pathways with subsequent effects on normal endocrine and metabolic functions [61,62]. More recently, research focused on changes in the epigenome and on inflammation.

Exposure during the prenatal period may result in epigenetic alterations modifying fetal programming and increasing, during postnatal life, the risk of some non-communicable diseases as suggested by the Developmental Origins of Health and Disease (DOHaD) hypothesis [38]. These changes can be caused by single EDCs (as previously mentioned) or by mixtures [63,64], which better represent real life, as demonstrated by the presence of many chemicals in umbilical cord blood.

Interestingly, single EDCs and mixtures might also interfere with the inflammasome during pregnancy. Associations between PBDE congeners and inflammation were described (although they did not reach statistical significance) in a representative USA sample from the National Health and Nutrition survey [65]. A significant association of first-trimester Interleukin (IL)-6 with environmental EDC exposure was described by Kelley et al. [66]. Interestingly, in our experience, increased IL-6 is associated with reduced IGF bioavailability in the placenta and fetal growth restriction [67], thus leading to a possible further pathogenetic mechanism that might not be entirely due to hypoxia, as originally hypothesized [67,68,69].

Finally, microRNAs were shown to be a regulatory network of upmost importance for all metabolic pathways and biological processes including growth [70]. MicroRNAs regulate gene expression and are well known to show changes in the presence of inflammation and in cancer [71]. Furthermore, there is building evidence that both factors during fetal life and exposure to EDCs facilitate the development of cancer in later life [72].

## 6. Prevention

Recently the International Federation of Gynecology and Obstetrics (FIGO) published an Opinion on Reproductive Health Impacts of Exposure to Toxic Environmental Chemicals [9]. Among the conclusions, the authors underlined that “*preventing exposure to environmental chemicals is a priority for reproductive health professionals everywhere*”. The Royal College of Obstetricians and Gynecologists, according to a “precautionary” approach to reducing the risk in the absence of causal evidence, concluded that “*despite uncertainty surrounding the effects of common environmental chemicals, mothers should be made aware of the sources and routes of exposure, the potential risks to the fetus/baby, and the important role that the mother can play in minimizing her baby’s chemical exposure*” [73].

These recommendations seem particularly significant because recent surveys showed some disappointing results. In the survey carried out by Rouillon et al., 54.3% of 300 French women who were interviewed during pregnancy or in the first days after the birth of their child had never heard about EDCs [74]. Little knowledge of the possible risks derived from contamination with EDCs was also reported in Canada [75]. Moreover, only a minority of medical doctors who follow pregnant mothers dedicate enough time to instructing the mothers on the prevention of the environmental risks and, in turn, they often feel culturally inadequate for this task [76]. It is, therefore, advisable that, in addition, to the necessary initiatives in both the legislative and public health programs, greater attention should be paid to the pre- and post-graduate training of doctors and, in particular, of obstetricians, with a need for awareness campaigns.

## 7. Conclusions and Perspectives

In summary, in recent years, a large number of epidemiological and animal studies (the latter were not taken into consideration in this mini-review) highlighted a relationship between exposure to EDCs, development of the placenta and fetal growth, and negative consequences on the state of health postnatally. For these reasons, international scientific societies recommended the implementation of all possible preventive measures through specific policies and education of health personnel and of the general population with particular emphasis on pregnant women [9,77]. Despite this, one cannot ignore the fact that the studies on the relationships between EDCs and fetal development often yielded heterogeneous results or did not show any statistical significance; however, the lack of statistical significance does not necessarily mean absence of an effect. This was seen in other areas of EDC studies. We agree with Trasande et al. when they write “*clearly, there are still many aspects of endocrine disruptor science that are unsettled, but these are generally related to the mechanisms themselves*” [78]. In other words, bearing in mind the complexity of a study that wants to demonstrate a more precise relationship of cause–effect between EDCs and fetal development, further research and new scientific approaches are needed. According to Kamai et al. who recently published a very thorough analysis of the literature on the effect of non-persistent chemicals (like phthalates and BPA) on fetal growth, many current studies are insufficiently powered or inadequately designed to detect effects [79]. Measurement of biomarkers (which, where, how often during pregnancy, in which population) and their possible cocktail effects, assessment of the outcome (when, correlated with gestational age, related to a prenatal ultrasonography follow-up), and a powerful statistical design are among the factors that may have influenced the lack of coherent conclusions until now [79].

In order to be able to define in more detail the relationship between exposure to endocrine disruptors during fetal life and the consequences on the development of the fetus and postnatal health, we still need not only well designed longitudinal research but the possibility of exploiting new techniques, particularly those integrating the exposome [80] with the different “omics” [81]. Finally, on the basis of an increasing amount of evidence, we cannot ignore that endocrine disruptors might interfere with the gut microbiome, contributing to the risk of developing some of the known chronic non-communicable diseases during the entire life span [82].
